# Harm reduction when it’s for our own kids: comment on Hagit Bonny-Noach, “The evolution of Israeli public policy for drug-using backpackers

**DOI:** 10.1186/s13584-018-0253-9

**Published:** 2018-10-24

**Authors:** Harold A. Pollack

**Affiliations:** Helen Ross Professor of Social Service Administration, University of Chicago, 969 East 60th Street, Chicago, IL 60637 USA

## Abstract

Hagit Bonny-Noach notes the challenging history of illicit substance use among Israeli backpackers. Few Israeli practices are more normative than the backpacking-trip as a rite of passage. Unsurprisingly, backpacking in far-off locales provides occasion to experiment with the various intoxicating experiences young adult life has to offer.

Some such experimentation is expected, and often developmentally appropriate. It also carries real risks and causes real harms. Israeli policymakers’ efforts to address this issue underscore both the necessity and the difficulty of harm reduction. Bonny-Noach usefully notes the importance of social class as both a facilitator and a barrier to effective public health policies to protect young adults from drug-related harms.

It is humbling as an American to comment on other nations’ health policies. Given our nation’s policy failures and pathologies, we often instruct by counter-example—and by illustrating how poor policies can produce poor population health outcomes despite the deployment of great human and material resources. This holds especially true of America’s policies towards intoxicating substances.

Still, a glance over the fence yields some consolation that other societies face their own challenges and worries. Such was my reaction to Hagit Bonny-Noach’s intriguing description of Israeli policies towards backpackers who use drugs [1].

Few Israeli practices are more normative than the backpacking-trip as a rite of passage. Few developments are less surprising than the news that backpacking in far-off locales provides occasion to experiment with the various intoxicating experiences young adult life has to offer.

Some such experimentation is expected, and often developmentally appropriate. It also carries real risks. Some of these risks are acute because they involve young men and women using powerful intoxicating substances for the very first time.

As elsewhere, the typical Israeli backpacker who uses hallucinogenics or other illicit substances will emerge unscathed. Carrying on with their lives, many might wonder why their parents and public health authorities are so concerned about the joint they might have smoked with friends by the campfire.

Yet an important minority will be tangibly harmed. Some of these harms are immediate: Overdose, sexual violence, unintended pregnancy, accidents and injury, toxic reactions to unfamiliar substances. Others, such as addiction disorders, unfold over time, but the harms are equally genuine for the minority of young adults, some with preexisting vulnerabilities, whose experiments with illicit substances go badly.

These risks are not unique to Israeli backpackers. Indeed these risks may be more pressing among others, such as young European tourists who frequent Mediterranean destinations with reputations for drinking, drug use, and night partying [2].

Such experimentation may also cast shadows on surrounding lives and communities. Much as Floridians welcome tourist dollars expended by college students over Spring Break, residents are less delighted by alcohol-related injuries and crime [3]. European tourist destinations such as Ibiza face similar community concerns [4]. One can imagine similar reactions among residents of Goa, India, where Israelis are important participants in the local drug scene.

Three other themes are noteworthy in this analysis.

## Harm reduction is both easier and harder when applied to normative behaviors among privileged groups than when it is applied to more-deviant behaviors among marginalized groups

Bonny-Noach notes the complex role of social class in defining feasible policy responses. Strictly punitive measures are out of the question when so many drug-using backpackers are members of Israel’s social and economic elite. These class dynamics bring more interesting implications, too.

Israelis appear relatively comfortable with syringe exchange and related services offered to people who inject drugs (PWID)—a distinctively more marginal group. HIV prevalence in Israel is relatively low. Harm reduction services are available in major cities. On Bonny-Noach’s account, this relatively humane response does not reflect particular regard for PWID within Israeli society. It may reflect the opposite. From the perspective of many decision-makers, PWID are already regarded as deviant and addicted. Thus, the population health calculus is straightforward and entirely pragmatic. If harm reduction measures reduce HIV incidence and pass the cost-benefit test, policymakers’ very psychological distance from marginalized drug-using communities makes harm reduction the sensible and pragmatic option.

Ironically enough, harm reduction can be more culturally fraught when equivocal messages and policies regarding behavioral risks are directed at “normative young adults” engaged in illicit substance use. Here the special circumstances of backpacking facilitates harm reduction interventions that might not be possible under other circumstances.

Fortunately, the mere fact that such drug use often occurs offshore—In India, East Asia, or central America-- softens the policy dilemma. Israel can offer treatment and prevention services to assist backpackers. Available law enforcement tools for supply-side and demand-side enforcement are correspondingly limited, allowing greater scope for harm reduction interventions such as the “Israeli Warm Home.”

## Destination venues may play a critical role in harm reduction

Hughes and colleagues plausibly suggest that unfamiliar drug markets, geographies, and climates heighten risks for both backpackers and destination communities. Backpackers arrive at relatively unfamiliar places, where they may not speak local languages, and are almost certain to lack basic information regarding medical services and harm reduction resources. The business models of destination hotels create further risks, as some venues attract tourists through their reputations for uninhibited partying and drug use.

Proper regulation by destination authorities--and proper interventions by Israeli public health authorities—might focus on effective partnerships with these venues.

Hotels, bars, and other sites can distribute basic information regarding drug-related risks and protective behaviors. They can also post information regarding prevention and treatment opportunities for individuals who need such help. The key is not to impose a zero-tolerance policy, but to ensure basic supports such as cold drinking water are available for those vulnerable to dehydration. The history of harm reduction measures at rave events provides one useful model.

## Certain harms—notably violence—appear notable in their absence

Benny-Noach presents 29 headlines from Israeli newspapers (see Table 1). These describe diverse substance-use-related problems associated with backpacking and substance use. Although illegal activity is mentioned in the context of Israeli or Indian police arresting drug dealers, none of these 29 headlines detailed violence perpetrated by or inflicted on Israeli backpackers. Israeli backpackers may, at times, pose a public nuisance within destination communities. They do not, generally, pose a public safety threat or appear to suffer disproportionate levels of predatory crime. This somewhat departs from the European or American experience, where violence is a more prominent drug-related harm.

These differences may reflect cross-national differences in alcohol use. Although alcohol disorders are more common among Israelis than stereotypes may suggest, prevalence is still relatively low by cross-national standards. [5] Within the European sample studied by Hughes and colleagues, more than 90% of reported violent incidents occurred when individuals were under the influence of alcohol. More than one-third of the European sample reported being drunk for at least half of their vacation stay. [2] Whatever the explanation, low prevalence of violence and of other non-drug crimes opens further space for less-punitive harm reduction measures.

## Conclusion

This is a valuable opportunity. Much can be accomplished when policymakers and the public are unafraid, when they see harm reduction as benefitting their own children and peers.

## Abbreviations

IADA: Israel Anti-Drug Authority
SCDAA: Special Committee on Drug and Alcohol Abuse
